# 1156. Pneumococcal Colonization in Children with Persistent Asthma and without Asthma

**DOI:** 10.1093/ofid/ofab466.1349

**Published:** 2021-12-04

**Authors:** Liset Olarte, Dithi Banerjee, Douglas S Swanson, Jennifer E Tabakh, Brian R Lee, Christopher J Harrison, Rangaraj Selvarangan

**Affiliations:** 1 Children’s Mercy Kansas City, Kansas City, Missouri; 2 Children’s mercy Hospital, Kansas City, MO; 3 Children’s Mercy Hospital, Kansas City, MO

## Abstract

**Background:**

The most common underlying medical condition among children ≥ 5 years of age with invasive pneumococcal disease is asthma. How asthma affects pneumococcal colonization is not fully understood. Our objective was to compare pneumococcal colonization rates in children with persistent asthma vs. without asthma.

**Methods:**

This is a single center retrospective cohort study. We used salvage mid-turbinate samples testing negative for influenza per routine care from 5-18 year-olds with upper respiratory symptoms or febrile illness during 2017-18 and 2018-19 northern hemisphere respiratory seasons (November to April). Analyzed groups were those with persistent asthma or those without asthma. Samples were evaluated for pneumococcal colonization by real-time PCR using CDC *lytA* primers (positive Ct ≤ 35). Positive samples were further tested with multiplex serotype-specific PCR assays to determine pneumococcal serotype.

**Results:**

Of 363 children (120 with persistent asthma and 243 without asthma), 87.6% were 5-10 years old; and 49.9% were male. Fifty percent of samples were from January-February. Pneumococcal colonization rate was lower in children with persistent asthma (10%) vs. without asthma (18.9%) (p=0.03). The odds of colonization were lower in children with persistent asthma (OR 0.4 [95%CI 0.2-0.9]) after adjusting for age, sex, clinic site, smoking exposure, and number of pneumococcal vaccine doses. Colonized patients without asthma were younger than the other groups (Table 1). Pneumococcal serotype/serogroup was assigned in 45 (77.6%) positive samples; 16 (36%) samples corresponded to PCV13 serotypes and 29 (64%) samples to non-PCV13 serotypes. The most common serotypes were: 19F (n=7), 3 (n=6), 6C/6D (n=5), 23B (n=4), 33F/33A/37 (n=4), 35B (n=3), 22F/22A (n=3), 23A (n=3).

Table 1

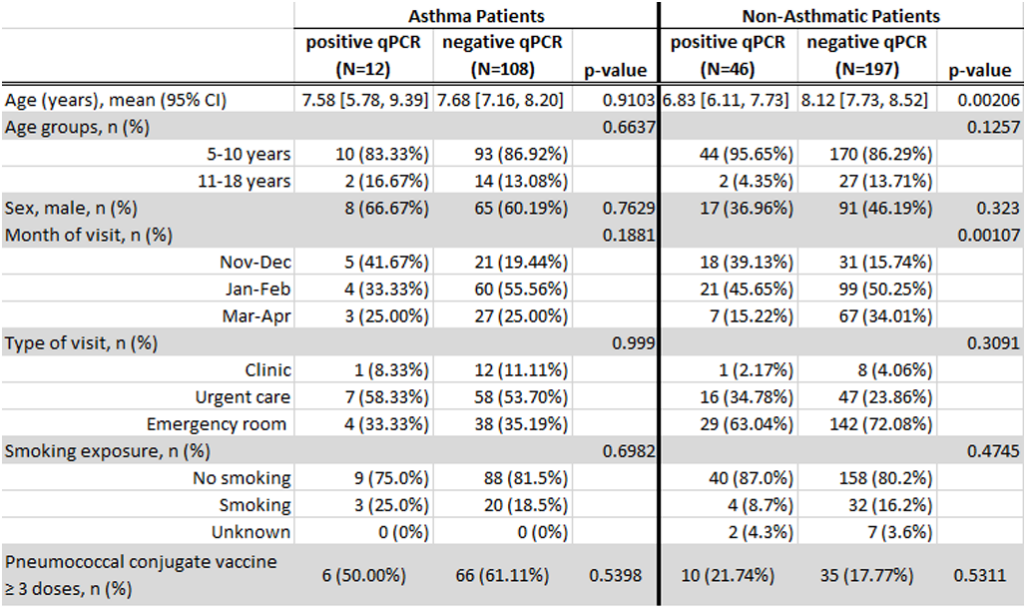

**Conclusion:**

Patients with persistent asthma had lower rates of pneumococcal colonization than patients without asthma during respiratory season.

**Disclosures:**

**Liset Olarte, MD, MSc**, **GSK** (Research Grant or Support)**Merck** (Research Grant or Support)**Pfizer** (Research Grant or Support)**Sanofi** (Research Grant or Support) **Douglas S. Swanson, MD**, **Merck** (Research Grant or Support)**Pfizer** (Research Grant or Support)**Sanofi** (Research Grant or Support) **Brian R. Lee, PhD, MPH** , **Merck** (Grant/Research Support)**Pfizer** (Grant/Research Support) **Christopher J. Harrison, MD**, **GSK** (Grant/Research Support)**Merck** (Grant/Research Support)**Pfizer** (Grant/Research Support, Scientific Research Study Investigator, Research Grant or Support)

